# Supplementary material for: Gene regulation by gonadal hormone receptors underlies brain sex differences
Source: Nature. 2022 May 4;606(7912):153–9. doi: 10.1038/s41586-022-04686-1 (PMC9159952; doi:10.1038/s41586-022-04686-1)
Supplement: Supplementary file 1 — Reporting Summary [file 41586_2022_4686_MOESM1_ESM.pdf]

nature research

Corresponding author(s): Jessica Tollkuhn

Last updated by author(s): Feb 13, 2022

Reporting Summary

Nature Research wishes to improve the reproducibility of the work that we publish. This form provides structure for consistency and transparency in reporting. For further information on Nature Research policies, see our Editorial Policies and the Editorial Policy Checklist.

Statistics

For all statistical analyses, confirm that the following items are present in the figure legend, table legend, main text, or Methods section.

n/a	Confirmed	
☐	☒	The exact sample size (*n*) for each experimental group/condition, given as a discrete number and unit of measurement
☐	☒	A statement on whether measurements were taken from distinct samples or whether the same sample was measured repeatedly
☐	☒	The statistical test(s) used AND whether they are one- or two-sided<br>*Only common tests should be described solely by name; describe more complex techniques in the Methods section.*
☐	☒	A description of all covariates tested
☐	☒	A description of any assumptions or corrections, such as tests of normality and adjustment for multiple comparisons
☐	☒	A full description of the statistical parameters including central tendency (e.g. means) or other basic estimates (e.g. regression coefficient) AND variation (e.g. standard deviation) or associated estimates of uncertainty (e.g. confidence intervals)
☐	☒	For null hypothesis testing, the test statistic (e.g. *F*, *t*, *r*) with confidence intervals, effect sizes, degrees of freedom and *P* value noted<br>*Give P values as exact values whenever suitable.*
☒	☐	For Bayesian analysis, information on the choice of priors and Markov chain Monte Carlo settings
☐	☒	For hierarchical and complex designs, identification of the appropriate level for tests and full reporting of outcomes
☒	☐	Estimates of effect sizes (e.g. Cohen's *d*, Pearson's *r*), indicating how they were calculated

Our web collection on statistics for biologists contains articles on many of the points above.

Software and code

Policy information about availability of computer code

Data collection

Adult and neonatal ATAC-seq: ATAC fastq files were processed using the ENCODE ATAC-seq pipeline (v1.7.0), with parameters: atac.auto_detect_adapter = TRUE, atac.multimapping = 0, atac.pval_thresh = 0.01. Bigwig tracks were generated from the filtered BAM files using deepTools (v3.5.0).

Adult, neonatal, and cell line CUT&RUN: Adapters and low-quality basecalls were trimmed using cutadapt (v2.8). Paired-end reads were aligned to mm10 using bowtie2 (v2.3.4.2). Duplicate reads were removed with picard (v2.18.20). Reads were filtered using SAMtools (v1.11). Peaks were called using MACS2 (v2.2.7.1). Bigwig tracks were generated using deepTools (v3.5.0).

Adult RiboTag RNA-seq: Adapters and low-quality basecalls were trimmed using fastx_toolkit (v0.0.13). Single-end reads were aligned to mm10 using STAR (v2.7.6). Reads overlapping gene exons were counted using Subread featureCounts (v2.0.1).

Neonatal bulk nuclear RNA-seq: Adapters and low-quality basecalls were trimmed using cutadapt (v2.8). Single-end reads were aligned to mm10 using STAR (v2.7.6). Technical duplicate reads (identical molecular identifier and read) were removed using nudup.py (v2.3.3). Reads overlapping each gene (including introns) were counted using Subread featureCounts (v2.0.1).

Single-nucleus multiome-seq: Raw sequencing data were processed using the Cell Ranger ARC pipeline (v2.0.0) with the cellranger-arc mm10 reference. Default parameters were used to align reads, count unique fragments or transcripts, and filter high-quality nuclei.

Single-nucleus RNA-seq: Raw sequencing data were processed using the Cell Ranger pipeline (v6.0.0) with the refdata-gex-mm10-2020-A reference. Default parameters were used to align reads, count unique transcripts, and filter high-quality nuclei.

Flow cytometry: Sony SH800S Cell Sorter Software (v2.1).

Data analysis	The following packages were used to analyze and visualize data: deepTools (v3.5.0), CUT&RUNTools scripts (https://bitbucket.org/qzhudfci/cutruntools/src/master/), MEME (v5.1.1), BEDTools (v2.29.0), clusterProfiler (v3.10.1), Gviz (v.1.34.1), DESeq2 (v1.30.1), DiffBind (v2.10.0), Seurat (v4.0.3), SingleCellExperiment (v1.12.0), ComplexHeatmap (v2.11.1), MetaNeighbor (v1.10.0), ChIPseeker (v1.18.0), eulerr (v6.1.1), ggplot2 (v3.3.3), pheatmap (v1.0.12), BETA (v1.0.7), seaborn (v0.11.0), pandas (v1.1.4), edgeR (v3.32.1), R stats (v4.0.3), chromVAR (v1.12.0), ArchR (v1.0.1), Signac (v1.3.0), regioneR (v1.22.0), Fiji/ImageJ (v2.0.0). Custom scripts can be found at https://github.com/gegenhu/estrogen_gene_reg.

For manuscripts utilizing custom algorithms or software that are central to the research but not yet described in published literature, software must be made available to editors and reviewers. We strongly encourage code deposition in a community repository (e.g. GitHub). See the Nature Research guidelines for submitting code & software for further information.

Data

Policy information about availability of data

All manuscripts must include a data availability statement. This statement should provide the following information, where applicable:

- Accession codes, unique identifiers, or web links for publicly available datasets
- A list of figures that have associated raw data
- A description of any restrictions on data availability

The data generated by this study are in GEO (GSE144718). Additional datasets were analyzed in this study: MCF7 ERa ChIP-seq (Franco et al. 2015, GSE59530), mouse liver 1 ERa ChIP-seq (Gertz et al. 2013, GSE49993), mouse liver 2 ERa ChIP-seq (Gordon et al. 2014, GSE52351), mouse uterus 1 ERa ChIP-seq (Hewitt et al. 2012, GSE36455), mouse uterus 2 ERa ChIP-seq (Gertz et al. 2013, GSE49993), mouse aorta ERa ChIP-seq (Gordon et al. 2014, GSE52351), mouse efferent ductules ERa ChIP-seq (Yao et al. 2017, Supplementary Info), mouse mammary gland ERa ChIP-seq (Palaniappan et al. 2019, GSE130032), mouse liver ATAC-seq (Cusanovich et al. 2018, GSE111586), BNST snRNA-seq (Welch et al. 2019, GSE126836), MPOA scRNA-seq (Moffitt et al. 2018, GSE113576), Allen Brain Institute Cell Type Database (Yao et al. 2020, https://portal.brain-map.org/atlases-and-data/rnaseq/mouse-whole-cortex-and-hippocampus-10x).

Field-specific reporting

Please select the one below that is the best fit for your research. If you are not sure, read the appropriate sections before making your selection.

☒ Life sciences ☐ Behavioural & social sciences ☐ Ecological, evolutionary & environmental sciences

For a reference copy of the document with all sections, see nature.com/documents/nr-reporting-summary-flat.pdf

Life sciences study design

All studies must disclose on these points even when the disclosure is negative.

Sample size	Sample sizes were determined by the following factors: 1) number of replicates previously used for ATAC-seq, RNA-seq, CUT&RUN, snRNA-seq, and multiome in the literature (Stroud et al., 2020, Neuron; Allaway et al., 2021; Nature; Di Bella et al., 2021, Nature), 2) cost of sequencing and animal maintenance, and 3) expected variability between samples for each assay after collecting preliminary data. For neonatal single-cell experiments, the target number of cells per sample was determined by prior estimates of cell type abundance from the published adult BNST snRNA-seq dataset (Welch et al., 2019, Cell).
Data exclusions	No data were excluded.
Replication	The number of biological replicates per condition for each experiment is listed below. All attempts at replication were successful.<br>MCF7 ERa CUT&RUN: n=2<br>Adult brain ERa CUT&RUN: n=2<br>Adult brain Nfix CUT&RUN: n=2<br>Adult gonadectomy+E2 ATAC-seq: n=3<br>Adult gonadally intact ATAC-seq: n=2<br>Adult RiboTag RNA-seq: n=4<br>Adult in situ hybridization: n=4<br>P14 Nfix immunofluorescent staining: n=6<br>Neonatal bulk nuclear RNA-seq: n=3<br>Neonatal ATAC-seq: n=3<br>Neonatal ERa CUT&RUN: n=2<br><br>For each sequencing experiment, brain tissue was pooled from 3-5 animals or 8-9 animals (adult RNA-seq only) per biological replicate to account for dissection variability. For the single-cell multiome experiments, brain tissue was pooled from 5 animals per condition prior to collection. For the P14 single-nucleus RNA-seq experiment, brain tissue was pooled from 3 animals per condition.
Randomization	For the adult treatment experiments, gonadectomized female and male mice were randomly assigned to vehicle and E2 groups. For the neonatal treatment experiments, females were randomly assigned to vehicle and E2 groups. For MCF-7 cell culture experiments, cells were randomly assigned to vehicle and E2 groups.
Blinding	Blinding was not performed for bioinformatics analysis, because knowledge of sample identity is required for designing statistical tests and visualizing data. Investigators were blinded to group allocation during data collection of bioinformatics experiments. For the ISH and IF experiments, the investigator was blinded during data collection and analysis.

Reporting for specific materials, systems and methods

We require information from authors about some types of materials, experimental systems and methods used in many studies. Here, indicate whether each material, system or method listed is relevant to your study. If you are not sure if a list item applies to your research, read the appropriate section before selecting a response.

Materials & experimental systems

n/a	Involved in the study
☐	☒ Antibodies
☐	☒ Eukaryotic cell lines
☒	☐ Palaeontology and archaeology
☐	☒ Animals and other organisms
☒	☐ Human research participants
☒	☐ Clinical data
☒	☐ Dual use research of concern

Methods

n/a	Involved in the study
☐	☒ ChIP-seq
☐	☒ Flow cytometry
☒	☐ MRI-based neuroimaging

Antibodies

Antibodies used	Human ERa CUT&RUN Ab #1: Santa Cruz sc-8002 (Lot 41718); Human ERa CUT&RUN Ab #2: Millipore Sigma 06-935 (Lot 2971020); Mouse ERa CUT&RUN Ab: Millipore Sigma 06-935 (Lot 2971020); Nfix CUT&RUN Ab: Abcam ab101341 (Lot GR3173994); Nfix IF Ab: Thermo Fisher PA5-30897 (Lot WA31716320); Guinea pig anti-rabbit IgG CUT&RUN Ab: Antibodies-Online ABIN101961 (Lot 42670); GFP IF Ab: Aves Labs GFP-1020 (Lot GFP697986); Anti-rabbit IgG-Cy3 Ab: Jackson Immuno 711-165-152; Anti-chicken IgG-488: Jackson Immuno 703-545-155.
Validation	All antibodies used for IF staining have been validated by the manufacturer for this purpose and have been used in prior publications (ERa IF: Wu et al. 2017; GFP IF: Mo et al. 2015; Nfix IF: Adam et al. 2020). The ERa antibody (Millipore Sigma 06-935) used for CUT&RUN was validated previously by IF staining in ERa knockout animals (Wu et al., 2017) and in this study through bioinformatic analysis, specifically 1) top enrichment of the ERE motif (Extended Data Fig. 1.1b, f), 2) similarity to prior ERa ChIP-seq data (Extended Data Fig. 1.1a-b, e-f), and 3) comparison between P0 brain Esr1+ and Esr1- cells (Fig. 3a). The Nfix antibody (Abcam ab101341) used for CUT&RUN was validated in this study in mHypoA cells and BNSTp by top enrichment of the NFI family motif (Extended Data Fig. 3.3i).

Eukaryotic cell lines

Policy information about cell lines

Cell line source(s)	MCF-7 cells - ATCC; mHypoA cells - Cedarlane Laboratories
Authentication	The human MCF-7 cell line was validated by STR profiling and morphology. The mouse mHypoA cell line was validated by morphology. Both cell lines were used only for preliminary validation of primary antibodies for CUT&RUN.
Mycoplasma contamination	Cell lines were not tested for mycoplasma contamination.
Commonly misidentified lines (See ICLAC register)	No commonly misidentified lines were used.

Animals and other organisms

Policy information about studies involving animals; ARRIVE guidelines recommended for reporting animal research

Laboratory animals	Esr1-Cre, Vgat-Cre, Rpl22-HA, Sun1-GFP, and C57Bl6/J wildtype mice were obtained from Jackson labs. Esr1-flox mice were received from Sohaib A. Khan.<br>Adult CUT&RUN experiments were performed on C57Bl6/J female and male mice at 8-12 weeks of age.<br>Adult RNA-seq and ISH experiments were performed on Esr1-Cre/Rpl22-HA female and male mice at 8-12 weeks of age.<br>Adult ATAC-seq experiments were performed on Esr1-Cre/Sun1-GFP female and male mice at 8-12 weeks of age.<br>Neonatal ATAC-seq, bulk RNA-seq (female only), IF staining, and multiome experiments were performed on Esr1-Cre/Sun1-GFP female and male mice at P4 and P14 (IF and multiome only).<br>Neonatal CUT&RUN was performed on Esr1-Cre/Sun1-GFP female mice at P0.<br>Neonatal snRNA-seq was performed on Vgat-Cre/Esr1-flox/Sun1-GFP female and male mice at P14.
Wild animals	None
Field-collected samples	None
Ethics oversight	All mouse experiments were performed under strict guidelines set forth by the CSHL Institutional Animal Care and Use Committee (IACUC).

Note that full information on the approval of the study protocol must also be provided in the manuscript.

ChIP-seq

Data deposition

☒ Confirm that both raw and final processed data have been deposited in a public database such as GEO.

☒ Confirm that you have deposited or provided access to graph files (e.g. BED files) for the called peaks.

Data access links<br>*May remain private before publication.*	CUT&RUN data are available in GEO (GSE144718). Reviewer access token is shapeowsfdwznot
Files in database submission	narrowPeak, bigwig, and raw fastq files are available for each sample.
Genome browser session<br>(e.g. UCSC)	Tracks can be visualized in a genome browser with the provided bigwig files.

Methodology

Replicates	MCF-7 ERa CUT&RUN, n=2 per treatment and per antibody<br>Adult brain IgG CUT&RUN, n=1 per treatment<br>Adult brain ERa CUT&RUN, n=2 per treatment and sex<br>mHypoA Nfix CUT&RUN, n=1<br>Adult BNSTp Nfix CUT&RUN, n=2 per treatment and sex<br>Neonatal brain ERa CUT&RUN, n=2 per treatment<br>Neonatal brain IgG CUT&RUN, n=1 per treatment
Sequencing depth	Read numbers indicate 1) total number of PE sequencing reads and 2) number of PE reads after BAM processing.<br><br>MCF-7 ERa Ab #1 Veh 1: 17128542 total; 2629779 processed<br>MCF-7 ERa Ab #1 E2 1: 18385942 total; 4593339 processed<br>MCF-7 ERa Ab #1 Veh 2: 14951660 total; 1915579 processed<br>MCF-7 ERa Ab #1 E2 2: 26172689 total; 7248734 processed<br>MCF-7 ERa Ab #2 Veh 1: 40779817 total; 11701045 processed<br>MCF-7 ERa Ab #2 E2 1: 42427425 total; 12165932 processed<br>MCF-7 ERa Ab #2 Veh 2: 39253805 total; 7674110 processed<br>MCF-7 ERa Ab #2 E2 2: 37248955 total; 9848088 processed<br>Adult brain IgG Veh 1: 17535876 total; 2873358 processed<br>Adult brain IgG E2 1: 16698729 total; 2521465 processed<br>Adult brain ERa male Veh 1: 20533002 total; 3293050 processed<br>Adult brain ERa male E2 1: 15185294 total; 2638242 processed<br>Adult brain ERa female Veh 1: 32037063 total; 5923281 processed<br>Adult brain ERa female E2 1: 31370254 total; 6464871 processed<br>Adult brain ERa male Veh 2: 23405701 total; 4078205 processed<br>Adult brain ERa male E2 2: 26406065 total; 4251286 processed<br>Adult brain ERa female Veh 2: 43085293 total; 7658066 processed<br>Adult brain ERa female E2 2: 41292689 total; 6021754 processed<br>P0 brain IgG Veh 1: 31842043 total; 9424613 processed<br>P0 brain IgG E2 1: 28725177 total; 8714349 processed<br>P0 brain ERa female Esr1+ Veh 1: 40857568 total; 8107696 processed<br>P0 brain ERa female Esr1+ E2 1: 39814592 total; 7438993 processed<br>P0 brain ERa female Esr1+ Veh 2: 39430462 total; 8574318 processed<br>P0 brain ERa female Esr1+ E2 2: 36778406 total; 5667825 processed<br>P0 brain ERa female Esr1- E2 1: 29611541 total; 8061354 processed<br>mHypoA Nfix: 10797210 total; 2778488 processed<br>Adult BNSTp Nfix male Veh 1: 23589255 total; 4300252 processed<br>Adult BNSTp Nfix male E2 1: 25583254 total; 5000254 processed<br>Adult BNSTp Nfix female Veh 1: 17477908 total; 4489675 processed<br>Adult BNSTp Nfix female E2 1: 19349717 total; 3705371 processed<br>Adult BNSTp Nfix male Veh 2: 22677910 total; 3543642 processed<br>Adult BNSTp Nfix male E2 2: 23858110 total; 3026763 processed<br>Adult BNSTp Nfix female Veh 2: 17843258 total; 4988121 processed<br>Adult BNSTp Nfix female E2 2: 21971104 total; 3185951 processed
Antibodies	Human ERa CUT&RUN Ab #1: Santa Cruz sc-8002 (Lot 41718); Human ERa CUT&RUN Ab #2: Millipore Sigma 06-935 (Lot 2971020); Mouse ERa CUT&RUN Ab: Millipore Sigma 06-935 (Lot 2971020); Nfix CUT&RUN Ab: Abcam ab101341 (Lot GR3173994); Guinea pig anti-rabbit IgG CUT&RUN Ab: Antibodies-Online ABIN101961 (Lot 42670).
Peak calling parameters	Peaks were called using MACS2 callpeak. Differential peaks were called using DiffBind, with specific parameters indicated in the Methods section.
Data quality	Only differential peaks that reached statistical significance were used for downstream analysis. For Nfix CUT&RUN, only peaks that were present across both sexes and treatment conditions were considered to be consensus peaks. For brain CUT&RUN experiments, target peaks overlapping peaks called in the IgG control were filtered out. Total peak numbers for each sample: MCF-7 ERa Ab #1 Veh 1: 610, MCF-7 ERa Ab #1 E2 1: 9506, MCF-7 ERa Ab #1 Veh 2: 642, MCF-7 ERa Ab #1 E2 2: 16153, MCF-7 ERa Ab #2 Veh 1: 11748,

MCF-7 ERa Ab #2 E2 1: 9331, MCF-7 ERa Ab #2 Veh 2: 4417, MCF-7 ERa Ab #2 E2 2: 32817, Adult brain ERa male Veh 1: 9719, Adult brain ERa male E2 1: 10143, Adult brain ERa female Veh 1: 5520, Adult brain ERa female E2 1: 4965, Adult brain ERa male Veh 2: 15900, Adult brain ERa male E2 2: 13533, Adult brain ERa female Veh 2: 2395, Adult brain ERa female E2 2: 3754, P0 brain ERa female Esr1+ Veh 1: 4, P0 brain ERa female Esr1+ E2 1: 10924, P0 brain ERa female Esr1+ Veh 2: 7, P0 brain ERa female Esr1+ E2 2: 9098, mHypoA Nfix: 30825, Adult BNSTp Nfix male Veh 1: 24801, Adult BNSTp Nfix male E2 1: 39528, Adult BNSTp Nfix female Veh 1: 49151, Adult BNSTp Nfix female E2 1: 63111, Adult BNSTp Nfix male Veh 2: 69995, Adult BNSTp Nfix male E2 2: 21358, Adult BNSTp Nfix female Veh 2: 62390, Adult BNSTp Nfix female E2 2: 63019.

Software

The following packages were used to analyze and visualize CUT&RUN data: deepTools (v3.5.0), CUT&RUNTools scripts (https://bitbucket.org/qzhudfci/cutruntools/src/master/), MEME (v5.1.1), BEDTools (v2.29.0), clusterProfiler (v3.10.1), Gviz (v.1.34.1), DiffBind (v2.10.0), edgeR (v3.32.1), ChIPseeker (v1.18.0), eulerr (v6.1.1), ggplot2 (v3.3.3), BETA (v1.0.7), seaborn (v0.11.0), pandas (v1.1.4).

Flow Cytometry

Plots

Confirm that:

☒ The axis labels state the marker and fluorochrome used (e.g. CD4-FITC).

☒ The axis scales are clearly visible. Include numbers along axes only for bottom left plot of group (a 'group' is an analysis of identical markers).

☒ All plots are contour plots with outliers or pseudocolor plots.

☒ A numerical value for number of cells or percentage (with statistics) is provided.

Methodology

Sample preparation

Esr1Cre/+; Sun1-GFPlx/+ mice (4 pooled per condition) were deeply anesthetized with ketamine/dexmedetomidine. 500-µm sections spanning the BNSTp were collected in a mouse brain matrix (Kent Scientific) on ice. The BNSTp was microdissected and collected in 1 ml of cold supplemented homogenization buffer (250 mM sucrose, 25 mM KCl, 5 mM MgCl2, 120 mM tricine-KOH, pH 7.8), containing 1 mM DTT, 0.15 mM spermine, 0.5 mM spermidine, and 1X EDTA-free PIC (Sigma Aldrich 11873580001). The tissue was dounce-homogenized 15x in a 1 ml glass tissue grinder (Wheaton) with a loose pestle. 0.3% IGEPAL CA-630 was added, and the suspension was homogenized 5x with a tight pestle. The homogenate was filtered through a 40-µm strainer then centrifuged at 500 x g for 15 min at 4oC. The pellet was resuspended in 0.5 ml homogenization buffer containing 1 mM DTT, 0.15 mM spermine, 0.5 mM spermidine, and 1X EDTA-free PIC. 30,000 GFP+ nuclei were collected into cold ATAC-RSB (10 mM Tris-HCl pH 7.5, 10 mM NaCl, 3 mM MgCl2) using the Sony SH800S Cell Sorter (purity mode) with a 100-µm sorting chip.

Instrument

Sony SH800S Cell Sorter

Software

Sony SH800S Cell Sorter Software

Cell population abundance

Esr1-Cre/Sun1-GFP+ population comprised ~10-20% of singlet nuclei. Vgat-Cre/Sun1-GFP+ population comprised ~40-60% of singlet nuclei.

Gating strategy

1) Nuclei were first gated on the BSC-A x FSC-A plot.
2) Singlet nuclei were gated on the linear axis of the FSC-H x FSC-A plot.
3) GFP+ singlet nuclei were gated on the FSC-A x GFP:EGFP-A plot.
Post-sort purity was assessed on a fluorescence microscope.

☒ Tick this box to confirm that a figure exemplifying the gating strategy is provided in the Supplementary Information.
